# Updated Australian category norms for neuropsychological and cognitive testing

**DOI:** 10.1080/00049530.2025.2607180

**Published:** 2026-01-12

**Authors:** Lidan Zheng, Brooke Brady, Ranmalee Eramudugolla, Kaarin J. Anstey

**Affiliations:** a School of Psychology, The University of New South Wales Sydney, Kensington, NSW, Australia; b Neuroscience Research Australia, Randwick, NSW, Australia; c UNSW Ageing Futures Institute, UNSW Sydney, Kensington, NSW, Australia

**Keywords:** Word list, category norms, category production, semantic fluency, lexical frequency, semantic typicality

## Abstract

**Objective:**

Category norms are lists of words commonly generated as belonging to given semantic categories and are routinely used in cognitive and neuropsychological assessments. While English-language norms from the US and UK are regularly updated, the only Australian norms were published more than three decades ago in 1988. This study aimed to update Australian category norms to facilitate culturally appropriate cognitive and neuropsychological assessment.

**Methods:**

Australian adults aged 18 to 85 years (*N* = 1011, M age = 52.67, SD = 18.89) completed a category production task. Participants were asked to list exemplars for 10 categories (Animal, Fruit, Sport, Clothing, Flower, Birds, Precious Stone, Furniture, Vehicle and Musical Instrument). Frequencies of words within each category were collated. Results were compared to 1988 Australian category norms and current UK category norms.

**Results:**

The members for each category occurring with a greater than 5% frequency are reported. Analyses showed that norms for Precious Stones were robust across time and region, while norms for Clothing and Birds were vulnerable to temporal and geographical shifts, respectively.

**Conclusions:**

These findings provide updated, context-relevant norms to inform both clinical and research-related neuropsychological and cognitive testing in Australia. They also demonstrate temporal, cultural and geographical variations in category norms.

## KEY POINTS

**What is already known about this topic:**

1. Category norms are lists of words belonging to a category. They are commonly used to create verbal learning tasks used in neuropsychological and cognitive assessments.
2. As category norms reflect context-specific taxonomies of concepts and semantics, they are likely to differ by region and change over time.
3. English norms derived from US and UK populations are regularly updated, but the most recent Australian category norms were published in 1988.

**What this topic adds:**

1. This study provides updated category norms for Australia which can be used to inform culturally specific psychology tasks for both clinical and research use.
2. We used a large, representative sample to generate norms for 10 widely used categories and compared differences in semantic fluency across time and geographical region.
3. The results reinforce the importance of using temporally relevant and culturally appropriate norms for psychological research.

## Introduction

Category production tasks are widely used in cognitive psychology and neuropsychology. In these tasks, participants are asked to name words as exemplars or members that belong to a given category, such as “Animals” or “Flowers”. The resulting word lists represent how frequently people produce specific exemplars for a given category and provide normative data (i.e., *category norms*) about how concepts are commonly represented in memory. These norms are used to investigate semantic memory, concept formation, and category typicality (Crowe & Prescott, 2003; Hampton & Gardiner, 1983; Ryan et al., 2008), as well as to develop experimental stimuli for studies of language, memory, and learning (Brandt, 1991; Elwood, 1995; Van Der Elst et al., 2005). Specifically, the data from category norms is often relied upon to choose the words for verbal learning and memory tests (e.g., Hopkins Verbal Learning Test (Brandt, 1991), California Verbal Learning Test (Elwood, 1995)) because they provide information about high frequency exemplars, lexical typicality and semantic clustering, reflecting how easily words are learned, recalled or confused.

The most widely cited and used set of category norms was published by Battig and Montague in 1969, who recruited 442 US students to generate exemplars for 56 categories. Since then, additional norms have been published both within the US and across other English-speaking countries, including the UK and Australia. The various category norms demonstrate that both cultural differences and temporal shifts can influence which exemplars are most salient within a given category. For example, a recently published paper compared UK and US norms and reported a correlation coefficient of r_s_ = 0.35 for production frequency (how often a word is listed in a category), indicating substantial regional differences. While some categories were relatively stable between countries (e.g., colours, precious stones), others (e.g., vehicles, sports) demonstrated environmental, cultural, and linguistic differences.

Currently, the only published Australian category norms were collected over 30 years ago by Casey and Heath (1988), who recruited 620 participants from both the University of Newcastle and the wider community in New South Wales. Most participants were under the age of 25 (83%). The study included 28 word categories, and participants were given 60 seconds to write down as many exemplars as they could think of for each category. The results reported the 20 most frequently produced words per category, along with their overall frequency and the frequency with which each was listed first. For example, in the category Birds, “Sparrow” was the most common response, produced 415 times in total and listed first by 80 participants.

The most recently published English-language category norms were collected by Banks and Connell (2023), who asked 60 students and staff from Lancaster University in the UK to generate word lists for 117 categories. They also compared production frequencies from their study with previous UK norms published in the mid-1980s (who also recruited their participants from universities, Hampton and Gardiner (1983)). The results revealed a shift in the lexicon over time across several categories (e.g., clothing, sport, fish). Overall, the correlations between the two datasets were around r_s_ = 0.45 for production frequency and r_s_ = 0.43 for first-rank frequency (the number of times a word is listed first in a given category), indicating limited stability in semantic exemplars over time, at least in the UK context. However, no recent Australian data are available to allow for a similar comparison.

The current study aims to generate an updated set of Australian category norms using a sample with a wide age range drawn from the general public in Australia. Many previously published norms, such as those cited above, relied heavily on young, university-based samples which were often drawn from a narrow geographic area (Banks & Connell, 2023; Battig & Montague, 1969; Casey & Heath, 1988). Such samples tend to lack demographic diversity and may not fully represent the category knowledge of the wider population. By collecting exemplar responses from a large and diverse sample of community-dwelling Australian participants recruited from around the country, we aim to provide a resource that reflects both universal category structures as well as informs understanding of temporal/historical shifts in lexicon over time and context-specific variations in exemplar typicality. This dataset will allow researchers working in Australia to design more ecologically valid experiments, to conduct cross-cultural investigations of semantic memory and conceptual organisation and can provide category norms for use in clinical assessments.

The specific aims of this study include: (1) Generating updated Australian norms for 10 widely used semantic categories, (2) Comparing these norms against older Australian norms published in Casey and Heath (1988) and UK norms published by Banks and Connell (2023) to identify any temporal and regional differences.

## Methods

### Participants

Australian participants aged 18–85 were invited to complete a 15-minute online task. Participants were recruited using Qualtrics panels with stratification for age and sex (*n* = 300 each from the following age groups evenly split by sex: 18–35, 36–55, 56–70, 71–85). Participants received up to $7 for their participation. This research was approved by the University of New South Wales Human Research Ethics Panel for the Behavioural Sciences (approval number: HC3592). All participants provided informed consent.

### Procedure

At the start of the task, participants were told: “The purpose of this task is to find out what objects or things people commonly think of as belonging to various groups or categories”. Participants were given 60 seconds to write down as many items as possible from each of 10 categories. To ensure that participants understood the task, they were given 30 seconds to do a practice task for the category of “Colour”. Participants were then asked to generate members from the following 10 target categories: “Animal”, “Fruit”, “Sport”, “Clothing”, “Flower”, “Precious Stone”, “Furniture”, “Vehicle”, “Bird” and “Musical Instrument”.

Compared to the 28 word categories included in the Casey and Heath (1988) study, the 10 categories included in this task were chosen because they were primarily concrete (rather than abstract, such as “Emotion” or “Religion”), relatively stable across time (excluding categories like “Famous Australian” or “Girl’s name”), and suitable for future psychological research (excluding potentially problematic categories such as “Crime”, “Drug”, or “Derogatory term”). With the exception of “Musical Instrument” (included because it was used in Banks and Connell (2023)), all selected categories overlapped with those in Casey and Heath (1988), allowing for direct comparisons across time.

### Data cleaning

The parameters for data cleaning were created iteratively upon data screening, guided by the conventions used in previous studies (Banks & Connell, 2023; Casey & Heath, 1988). The data were cleaned by LZ and involved correcting spellings, collapsing singular words and plurals, collapsing words that mean the same thing (e.g., Bike, Bicycle), removing nonsense answers and removing incorrect answers (e.g., brand names or associated words). For transparency, both the raw and cleaned data are available via the OSF repository.

### Analysis

In line with Casey and Heath (1988) and Banks and Connell (2023), Table 1 provides descriptions of the variables that were extracted for each category, including category size, mean number of responses per category, production frequency for each item, mean rank for each item, first rank frequency for each item and best category exemplars.

**Table 1.** Variables included in category norms.

Category Measures	
Category size	The total number of unique, non-idiosyncratic member words (i.e., members produced by more than one participant) that were listed for a given category across the entire set of participants
Mean number of responses	The average number of responses produced by each participant for a given category, calculated as the total number of non-idiosyncratic responses collated for that category divided by the number of participants who saw that category
Item level	
Production frequency	The number of participants who named a particular member word within its category
Mean rank	The mean ordinal position of a particular member word within its category
First rank frequency	The number of participants who named a particular member first within its category
Best category exemplars	Top 2 members of the category that people report first (i.e., first response that comes to mind for a given category)

### Comparisons across time and region

We compared our production frequencies for each category to those reported in Casey and Heath (1988) and Banks and Connell (2023) to assess similarities across time (2020s vs 1980s in Australia) and regions (2020s Australian vs 2020s UK norms). To enable direct comparison across datasets, we first identified all items that appeared in both lists for a given category. Spearman’s correlations were calculated for each set of matching pairs of words (N pairs) with ≥5% frequency in each category. The referential version for each category (i.e., the version that collapsed across words that mean the same thing) reported by Banks and Connell (2023) was used for comparison.

Because production frequency was not normally distributed, Spearman’s rank correlations were used instead of Pearson’s correlations (Banks & Connell, 2023). In psychology, a correlation coefficient between 0.1 and 0.3 is generally considered a weak effect, between 0.3 and 0.5 is a moderate effect and above 0.5 is a strong effect (Cohen, 1988).

As all analyses were descriptive rather than inferential (i.e., no hypothesis tests were conducted) and correlations were only used to characterise patterns of similarity across datasets, no corrections for multiple comparisons were required in this study.

## Results

A total of 1422 people opened the survey link in March 2022. Data were screened and cases were removed if they were incomplete or deemed to be unreliable due to unrealistically fast response times (less than half the median time), duplicate responses or responding that was indicative of bots (using Google’s invisible reCAPTCHA technology). The final sample included 1011 adults with complete data for analyses. We had representation from every state and territory in Australia. The mean age of the participants was 52.67 years with a roughly even distribution across age brackets, males and females and socioeconomic brackets (see Table 2 for demographics).

**Table 2.** Participant demographics.

Mean age (SD)	52.67 (18.89)
Age distribution	
18–35	252 (25%)
36–55	250 (25%)
56–70	280 (28%)
71–85	229 (23%)
Mean years of education (SD)	14.04 (6.13)
Sex (%)	
Male	667 (52%)
Female	606 (47%)
Intersex	1 (0.08%)
Prefer not to say	2 (0.16%)
State/Territory	
NSW	288 (28%)
VIC	235 (23%)
QLD	230 (23%)
SA	109 (11%)
WA	94 (9%)
TAS	37 (4%)
ACT	11 (1%)
NT	3 (<1%)
Missing	4 (<1%)
SES^a^	
High (SEIFA 8–10)	322
Medium (SEIFA 4–7)	438
Low (SEIFA 1–3)	240

^a^Participant socioeconomic status (SES) was determined based on the provided postcode and categorised using the Australian Socio-Economic Indexes for Areas (SEIFA).

### Category norms

Table 3 shows the summary statistics for each category. The largest category (where participants generated the highest number of unique responses) was Furniture (376 unique answers) and the smallest category was Fruit (113 unique answers). The largest trimmed category (excluding items with < 5% response frequency) was Animals (48 unique answers) and the smallest trimmed category was Precious stones (16 unique answers).

**Table 3.** Category norms summary.

Category	N (total participants)	Category size (untrimmed)	Trimmed category size (exclude items with <5% response rate)	Mean number of responses	Best category exemplars
Animal	977	280	48	11.45	Dog, Cat
Bird	934	214	40	8.07	Parrot, Eagle
Clothing	928	307	35	10.20	Shirt, Dress
Flower	947	249	26	5.80	Rose, Daisy
Fruit	982	113	31	9.66	Apple, Banana
Furniture	955	376	32	7.95	Chair, Table
Precious Stone	880	168	16	6.00	Diamond, Opal
Musical Instrument	978	165	31	8.34	Guitar, Piano
Sport	988	245	35	9.04	Football, Cricket
Vehicle	779	309	23	7.87	Car, Truck

The final list of category members occurring with ≥5% response frequency is provided in the Supplementary File. For items that are abbreviations or refer to the same thing (e.g., drums and drum kit), the less frequently reported spelling is given in brackets. Responses that were closely related but did not refer to the same core referent were maintained as separate items (e.g., Couch and Sofa).

### Comparisons across time and region

Table 4 shows the cross-temporal and cross-regional correlations for production frequency for each category. Correlations could only be completed for category members that occurred on both lists (this number is denoted by N pairs).

**Table 4.** Temporal and regional comparison of category norms using Spearman’s correlation.

Category	1980s Australian Norms (Casey & Heath)^a^		2020s UK Norms (Banks & Connell)	
	N pairs	Correlation (r_s_)	N pairs	Correlation (r_s_)
Animals	20	.870**	43	.725**
Birds	20	.675**	29	.428*
Clothing	20	.487*	29	.547**
Flowers	17	.710**	16	.762**
Fruit	19	.714**	22	.724**
Furniture	17	.783**	21	.808**
Precious Stones	16	.965**	13	.916**
Musical Instruments^b^	NA	NA	28	.858**
Sports	19	.777**	30	.784**
Vehicle	12	.888**	15	.556*

^a^Casey & Heath only reported the top 20 items for each category.

^b^Casey & Heath did not include musical instruments in their study.

**p < .01, *p < .05.

Compared to 1980s Australian norms, the category that remained the most similar was Precious stones (r_s_ = 0.956), and the category that was the most dissimilar was Clothing (r_s_ = 0.487). Compared to UK norms, the category that was most similar was also Precious stones (r_s_ = 0.916), and the category that was the most dissimilar was Birds (r_s_ = 0.428).

## Discussion

Overall, our results bear similarities to the Casey and Heath (1988) and Banks and Connell (2023) studies but demonstrate distinct differences related to time and region (country). Our largest trimmed category (i.e., excluding items that were generated by less than 5% of participants as category exemplars) was Animals which mimicked both comparative studies. Our smallest trimmed category was Precious stones, which, out of the categories included in this study, was also the smallest in Casey and Heath (1988) and Banks and Connell (2023).

Our correlation analysis showed that compared to 1980s Australian and 2020s UK data, the category that had the highest correlation with both sets of norms was Precious Stones. This indicates that the norms for this category are cross-culturally and cross-temporally robust. This is likely because precious stones consist of a small, highly prototypical set of exemplars (e.g., diamond, ruby, sapphire, opal) where there is little opportunity for new items to enter the category, resulting in minimal variability across samples and time.

The category that had the smallest correlation (i.e., the one that had changed the most) from 1980s Australian data was Clothing, indicating that this category is highly sensitive to cultural, social, and generational change, including temporal shifts in fashion trends, social norms and clothing terminology (e.g., “Shorts” was 5th most frequently reported item of clothing in this study, whereas it ranked 16th in the Casey and Heath (1988) study).

The category that had the smallest correlation with 2020 UK data was Birds, indicating that this category varies substantially by context. This is likely because of geographical differences in native fauna which shapes the types of birds that people see in their daily lives. For example, the second most reported bird in the Banks and Connell (2023) study was “Blue Tit” which did not appear at all on the Australian list.

Overall, our results suggest that category norms evolve in ways that mirror cultural trends, environmental exposure, and the stability of underlying conceptual structures. The differences observed across time and regions reinforce the value of updating category norms periodically and underscore the importance of considering cultural and ecological context when comparing or applying such norms across populations.

### Strengths and limitations

The strengths of this study over previous research are that it included a large sample recruited from around Australia that was stratified by age and sex (compared to Casey and Heath (1988) who primarily recruited young participants from within NSW). This means our norms are likely more representative of a wider population of Australians than previous norms (Casey & Heath, 1988). However, we acknowledge that our sample is not necessarily nationally representative as we recruited solely using Qualtrics panels who consist of people who regularly participate in online surveys.

## Conclusion

As category norms serve as the foundation for many psychological assessments (e.g., verbal learning tests), it is essential to maintain their validity by ensuring they accurately represent the current semantic knowledge of the target population. This study presents an updated set of Australian category norms for use in future psychological research and practice. Our cross-temporal and cross-regional comparisons reinforce the importance of using up-to-date, context-specific norms to preserve the soundness of psychological tasks and highlight the need for ongoing work tracking how category production shifts across demographic groups and over time.

## Supplementary Material

Supplemental material

## Data Availability

The data that support the findings of this study are openly available on OSF at https://osf.io/3s6ym/overview?view_only=7a2df81bb5944406b161902d92b58b77.
