# Supplementary material for: Updated Australian category norms for neuropsychological and cognitive testing
Source: Aust J Psychol. 2026 Jan 12;78(1):2607180. doi: 10.1080/00049530.2025.2607180 (PMC12798662; doi:10.1080/00049530.2025.2607180)
Supplement: Supplemental material [file RAUP_A_2607180_SM0291.docx]

# **Supplementary File**

# Animals

Total participants (who produced correct answers): 977

Category size (ie unique answers): 280

Mean number of responses (ie unique answers/total participants): 11.45

Best category exemplars (italicised): Dog, Cat

| **Animal** | **Count** | **Production Frequency** | **Mean Rank** | **Number First** |
| --- | --- | --- | --- | --- |
| *Cat* | *930* | *95%* | *2.52* | *227* |
| *Dog* | *928* | *95%* | *2.06* | *462* |
| Lion | 559 | 57% | 6.05 | 43 |
| Tiger | 524 | 54% | 6.29 | 35 |
| Horse | 457 | 47% | 6.08 | 32 |
| Elephant | 417 | 43% | 6.73 | 18 |
| Cow | 404 | 41% | 6.46 | 30 |
| Bird | 364 | 37% | 5.73 | 19 |
| Giraffe | 284 | 29% | 8.41 | 7 |
| Sheep | 258 | 26% | 8.31 | 2 |
| Pig | 252 | 26% | 7.53 | 6 |
| Fish | 235 | 24% | 7.23 | 1 |
| Monkey | 230 | 24% | 7.83 | 7 |
| Mouse | 226 | 23% | 6.87 | 2 |
| Snake | 226 | 23% | 8.99 | 0 |
| Kangaroo | 200 | 20% | 9.20 | 5 |
| Zebra | 186 | 19% | 7.76 | 4 |
| Bear | 180 | 18% | 6.66 | 25 |
| Rabbit | 179 | 18% | 7.09 | 1 |
| Rat | 160 | 16% | 8.04 | 2 |
| Koala | 151 | 15% | 9.26 | 6 |
| Chicken | 148 | 15% | 8.35 | 0 |
| Goat | 143 | 15% | 8.29 | 3 |
| Lizard | 114 | 12% | 9.09 | 1 |
| Donkey | 111 | 11% | 8.30 | 0 |
| Rhinoceros | 104 | 11% | 9.91 | 0 |
| Hippopotamus | 86 | 9% | 10.29 | 0 |
| Cheetah | 80 | 8% | 9.44 | 1 |
| Fox | 79 | 8% | 8.59 | 3 |
| Leopard | 79 | 8% | 8.81 | 0 |
| Frog | 78 | 8% | 7.64 | 5 |
| Shark | 77 | 8% | 10.51 | 1 |
| Crocodile | 75 | 8% | 9.87 | 0 |
| Guinea pig | 74 | 8% | 8.99 | 1 |
| Wolf | 74 | 8% | 7.53 | 2 |
| Camel | 73 | 7% | 7.84 | 0 |
| Wombat | 72 | 7% | 9.43 | 0 |
| Gorilla | 71 | 7% | 9.21 | 1 |
| Panda | 71 | 7% | 8.70 | 2 |
| Duck | 70 | 7% | 8.04 | 4 |
| Possum | 64 | 7% | 8.92 | 0 |
| Deer | 63 | 6% | 9.08 | 2 |
| Turtle | 63 | 6% | 9.25 | 0 |
| Dolphin | 59 | 6% | 11.12 | 1 |
| Whale | 59 | 6% | 11.10 | 0 |
| Ape | 58 | 6% | 9.28 | 1 |
| Emu | 54 | 6% | 11.17 | 0 |
| Dingo | 52 | 5% | 8.50 | 0 |

# Fruit

Total participants: 982

Category size: 113

Mean number of responses: 9.66

Best category exemplars (italicised): Apple, Banana

| **Fruit** | **Count** | **Production Frequency** | **Mean Rank** | **Number First** |
| --- | --- | --- | --- | --- |
| *Apple* | *942* | *96%* | *1.86* | *598* |
| Orange | 769 | 78% | 3.68 | 97 |
| *Banana* | *763* | *78%* | *3.72* | *136* |
| Grape | 583 | 59% | 5.60 | 18 |
| Pear | 561 | 57% | 4.37 | 27 |
| Peach | 432 | 44% | 6.66 | 10 |
| Strawberry | 430 | 44% | 6.37 | 15 |
| Plum | 336 | 34% | 7.08 | 3 |
| Watermelon | 330 | 34% | 6.90 | 9 |
| Mango | 329 | 34% | 6.21 | 17 |
| Blueberry | 280 | 29% | 8.04 | 1 |
| Kiwi fruit | 279 | 28% | 6.68 | 10 |
| Lemon | 270 | 27% | 6.69 | 11 |
| Mandarin | 255 | 26% | 7.29 | 1 |
| Nectarine | 244 | 25% | 8.30 | 2 |
| Pineapple | 215 | 22% | 6.63 | 5 |
| Apricot | 208 | 21% | 7.14 | 2 |
| Raspberry | 205 | 21% | 8.15 | 1 |
| Tomato | 173 | 18% | 7.70 | 1 |
| Cherry | 168 | 17% | 7.09 | 2 |
| Rockmelon | 145 | 15% | 9.01 | 0 |
| Lime | 139 | 14% | 8.16 | 3 |
| Blackberry | 136 | 14% | 8.74 | 1 |
| Grapefruit | 126 | 13% | 6.90 | 1 |
| Passionfruit | 103 | 10% | 8.30 | 1 |
| Dragonfruit | 88 | 9% | 9.00 | 1 |
| Melon | 86 | 9% | 7.44 | 0 |
| Avocado | 85 | 9% | 7.93 | 1 |
| Fig | 70 | 7% | 8.16 | 0 |
| Lychee | 49 | 5% | 10.06 | 0 |
| Honeydew melon | 48 | 5% | 9.60 | 0 |

# Sports

Total participants: 988

Category size: 245

Mean number of responses: 9.04

Best category exemplars (italicised): Football, Cricket

| **Sport** | **Count** | **Production Frequency** | **Mean Rank** | **Number First** |
| --- | --- | --- | --- | --- |
| Tennis | 704 | 71% | 4.19 | 131 |
| Soccer | 677 | 69% | 3.48 | 129 |
| *Football* | *630* | *64%* | *2.73* | *237* |
| *Cricket* | *532* | *54%* | *3.83* | *138* |
| Basketball | 460 | 47% | 5.13 | 30 |
| Swimming | 457 | 46% | 6.25 | 14 |
| Rugby | 382 | 39% | 4.14 | 57 |
| Golf | 306 | 31% | 5.12 | 33 |
| Netball | 295 | 30% | 5.69 | 21 |
| Baseball | 290 | 29% | 5.82 | 14 |
| Hockey | 286 | 29% | 5.76 | 30 |
| AFL | 220 | 22% | 4.27 | 49 |
| Running | 211 | 21% | 7.07 | 9 |
| Badminton | 198 | 20% | 6.68 | 9 |
| Volleyball | 156 | 16% | 6.71 | 1 |
| Skiing | 140 | 14% | 8.37 | 0 |
| Squash | 117 | 12% | 6.86 | 0 |
| Athletics | 115 | 12% | 7.14 | 3 |
| Rugby league | 107 | 11% | 3.73 | 22 |
| Table tennis | 96 | 10% | 6.78 | 1 |
| Boxing | 87 | 9% | 7.80 | 2 |
| Gymnastics | 87 | 9% | 8.48 | 1 |
| Softball | 87 | 9% | 7.03 | 0 |
| Rugby union | 83 | 8% | 4.34 | 9 |
| Cycling | 70 | 7% | 7.73 | 1 |
| Surfing | 65 | 7% | 7.28 | 2 |
| NRL | 62 | 6% | 4.94 | 2 |
| Polo | 56 | 6% | 7.13 | 1 |
| Diving | 55 | 6% | 9.93 | 0 |
| Wrestling | 52 | 5% | 8.37 | 1 |
| Lacrosse | 49 | 5% | 8.12 | 0 |
| Ice hockey | 48 | 5% | 8.04 | 0 |
| Rowing | 48 | 5% | 8.17 | 0 |
| Archery | 47 | 5% | 8.32 | 0 |
| Bowls | 47 | 5% | 6.81 | 2 |

# Clothes

Total participants: 928

Category size: 307

Mean number of responses: 10.20

Best category exemplars (italicised): Shirt, Dress

| **Clothes** | **Count** | **Count** | **Production Frequency** | **Mean Rank** |
| --- | --- | --- | --- | --- |
| *Shirt* | *699* | *75%* | *2.81* | *291* |
| Socks | 510 | 55% | 6.50 | 13 |
| Pants | 508 | 55% | 3.31 | 80 |
| *Dress* | *489* | *53%* | *4.55* | *132* |
| Shorts | 476 | 51% | 5.35 | 29 |
| Skirt | 417 | 45% | 5.28 | 17 |
| Underwear | 415 | 45% | 6.42 | 6 |
| Jumper | 383 | 41% | 6.32 | 23 |
| Jacket | 381 | 41% | 7.09 | 15 |
| Jeans | 378 | 41% | 4.84 | 67 |
| T shirt | 349 | 38% | 4.65 | 74 |
| Trousers | 290 | 31% | 4.59 | 20 |
| Coat | 269 | 29% | 6.78 | 17 |
| Hat | 263 | 28% | 7.16 | 37 |
| Bra | 262 | 28% | 7.48 | 4 |
| Blouse | 227 | 24% | 5.70 | 8 |
| Shoes | 224 | 24% | 6.83 | 22 |
| Singlet | 223 | 24% | 7.03 | 7 |
| Scarf | 159 | 17% | 8.57 | 2 |
| Tie | 146 | 16% | 7.68 | 1 |
| Cardigan | 136 | 15% | 8.60 | 0 |
| Suit | 120 | 13% | 7.73 | 9 |
| Top | 106 | 11% | 3.51 | 29 |
| Vest | 99 | 11% | 8.05 | 1 |
| Gloves | 91 | 10% | 10.15 | 0 |
| Pajamas | 75 | 8% | 8.75 | 2 |
| Sweater | 70 | 8% | 7.11 | 2 |
| Hoodie | 66 | 7% | 8.58 | 1 |
| Leggings | 66 | 7% | 9.35 | 0 |
| Stockings | 54 | 6% | 9.33 | 0 |
| Slacks | 52 | 6% | 6.67 | 1 |
| Belt | 48 | 5% | 9.88 | 0 |
| Blazer | 45 | 5% | 8.89 | 0 |
| Tights | 45 | 5% | 9.96 | 0 |
| Polo shirt | 44 | 5% | 7.66 | 1 |

# Flower

Total participants: 947

Category size: 249

Mean number of responses: 5.80

Best category exemplars (italicised): Rose, Daisy

| **Flower** | **Count** | **Production Frequency** | **Mean Rank** | **Number First** |
| --- | --- | --- | --- | --- |
| *Rose* | *928* | *98%* | *1.68* | *624* |
| *Daisy* | *457* | *48%* | *3.37* | *105* |
| Lily | 355 | 38% | 4.24 | 22 |
| Tulip | 319 | 34% | 3.76 | 18 |
| Daffodil | 304 | 32% | 3.72 | 18 |
| Sunflower | 277 | 29% | 4.02 | 28 |
| Carnation | 208 | 22% | 4.09 | 6 |
| Pansy | 171 | 18% | 3.89 | 15 |
| Orchid | 144 | 15% | 4.64 | 5 |
| Violet | 109 | 12% | 4.51 | 6 |
| Petunia | 102 | 11% | 4.50 | 6 |
| Iris | 90 | 10% | 4.52 | 10 |
| Chrysanthemum | 89 | 9% | 4.62 | 5 |
| Gerbera | 87 | 9% | 4.28 | 6 |
| Geranium | 83 | 9% | 4.33 | 3 |
| Poppy | 80 | 8% | 4.70 | 6 |
| Lavender | 78 | 8% | 5.26 | 0 |
| Frangipani | 72 | 8% | 4.64 | 2 |
| Hibiscus | 70 | 7% | 4.69 | 7 |
| Dahlia | 70 | 7% | 4.53 | 5 |
| Jasmine | 55 | 6% | 4.45 | 2 |
| Peony | 55 | 6% | 5.22 | 1 |
| Hydrangea | 53 | 6% | 5.21 | 3 |
| Dandelion | 52 | 5% | 4.60 | 4 |
| Marigold | 51 | 5% | 5.12 | 1 |
| Gardenia | 48 | 5% | 4.63 | 2 |

# Precious Stone

Total participants: 880

Category size: 168

Mean number of responses: 6.00

Best category exemplars (italicised): Diamond, Opal

| **Precious Stone** | **Count** | **Production Frequency** | **Mean Rank** | **Number First** |
| --- | --- | --- | --- | --- |
| *Diamond* | *868* | *99%* | *2.036866* | *423* |
| Ruby | 689 | 78% | 2.986938 | 115 |
| Sapphire | 506 | 58% | 3.960474 | 10 |
| *Opal* | *503* | *57%* | *3.477137* | *128* |
| Emerald | 494 | 56% | 3.508097 | 63 |
| Amethyst | 250 | 28% | 4.592 | 20 |
| Pearl | 207 | 24% | 4.516908 | 6 |
| Gold | 180 | 20% | 3.611111 | 34 |
| Topaz | 170 | 19% | 5.117647 | 8 |
| Jade | 147 | 17% | 4.816327 | 4 |
| Garnet | 141 | 16% | 5.070922 | 2 |
| Quartz | 132 | 15% | 5.060606 | 9 |
| Silver | 108 | 12% | 4.703704 | 0 |
| Crystal | 74 | 8% | 3.959459 | 12 |
| Onyx | 59 | 7% | 5.813559 | 2 |
| Aquamarine | 53 | 6% | 6.169811 | 0 |

# Furniture

Total participants: 955

Category size: 376

Mean number of responses: 7.95

Best category exemplars (italicised): Chair, Table

| **Furniture** | **Count** | **Production Frequency** | **Mean Rank** | **Number First** |
| --- | --- | --- | --- | --- |
| *Chair* | *872* | *91%* | *2.27* | *335* |
| *Table* | *830* | *87%* | *2.72* | *204* |
| Bed | 670 | 70% | 4.32 | 73 |
| Desk | 421 | 44% | 4.39 | 77 |
| Lounge | 330 | 35% | 3.77 | 49 |
| Couch | 312 | 33% | 3.25 | 101 |
| Sofa | 303 | 32% | 3.87 | 43 |
| Coffee table | 252 | 26% | 6.32 | 3 |
| Wardrobe | 228 | 24% | 6.31 | 3 |
| Cupboard | 214 | 22% | 5.98 | 5 |
| Stool | 209 | 22% | 5.91 | 2 |
| Cabinet | 145 | 15% | 6.72 | 1 |
| Dresser | 116 | 12% | 6.40 | 0 |
| Drawers | 110 | 12% | 6.75 | 3 |
| Bedside table | 96 | 10% | 7.49 | 0 |
| Dining table | 92 | 10% | 5.73 | 1 |
| Bench | 88 | 9% | 6.42 | 0 |
| Bookcase | 87 | 9% | 7.00 | 1 |
| Side table | 86 | 9% | 6.77 | 1 |
| Recliner | 83 | 9% | 6.17 | 1 |
| Ottoman | 74 | 8% | 7.47 | 0 |
| Armchair | 71 | 7% | 6.14 | 2 |
| Bookshelf | 69 | 7% | 6.91 | 0 |
| Lamp | 64 | 7% | 6.97 | 0 |
| TV (Television) | 63 | 7% | 6.46 | 3 |
| Sideboard | 63 | 7% | 6.24 | 0 |
| TV unit | 58 | 6% | 6.74 | 0 |
| Shelf | 57 | 6% | 7.93 | 0 |
| Tallboy | 56 | 6% | 7.68 | 1 |
| Buffet | 55 | 6% | 6.96 | 0 |
| Chest of drawers | 54 | 6% | 7.70 | 0 |
| Seat | 47 | 5% | 4.62 | 6 |

# Vehicle

Total participants: 779

Category size: 309

Mean number of responses: 7.87

Best category exemplars (italicised): Car, Truck

| **Vehicle** | **Count** | **Production Frequency** | **Mean Rank** | **Number First** |
| --- | --- | --- | --- | --- |
| *Car* | *692* | *89%* | *1.31* | *605* |
| *Truck* | *566* | *73%* | *3.51* | *42* |
| Bus | 460 | 59% | 4.10 | 16 |
| Motorcycle | 413 | 53% | 4.63 | 7 |
| Bike  (Bicycle) | 333 | 43% | 4.78 | 4 |
| Train | 290 | 37% | 5.20 | 0 |
| Van | 233 | 30% | 5.10 | 6 |
| Airplane | 220 | 28% | 6.54 | 0 |
| Ute | 216 | 28% | 4.76 | 13 |
| Scooter | 197 | 25% | 6.39 | 0 |
| Boat | 167 | 21% | 5.89 | 1 |
| Sedan | 147 | 19% | 3.72 | 40 |
| SUV | 134 | 17% | 4.49 | 13 |
| Tram | 132 | 17% | 6.38 | 0 |
| 4 wheel drive | 106 | 14% | 4.88 | 7 |
| Tractor | 81 | 10% | 5.33 | 2 |
| Hatchback | 71 | 9% | 4.97 | 6 |
| Helicopter | 67 | 9% | 7.96 | 0 |
| Taxi | 64 | 8% | 6.56 | 0 |
| Semi trailer | 52 | 7% | 5.87 | 0 |
| Ship | 44 | 6% | 7.70 | 0 |
| Wagon | 43 | 6% | 6.30 | 0 |
| Lorry | 38 | 5% | 5.95 | 0 |

# Birds

Total participants: 934

Category size: 214

Mean number of responses: 8.07

Best category exemplars (italicised): Parrot, Eagle

| **Bird** | **Count** | **Production Frequency** | **Mean Rank** | **Number First** |
| --- | --- | --- | --- | --- |
| *Eagle* | *467* | *50%* | *4.48* | *88* |
| Magpie | 444 | 48% | 4.03 | 79 |
| *Parrot* | *399* | *43%* | *3.99* | *104* |
| Crow | 358 | 38% | 4.79 | 37 |
| Pigeon | 319 | 34% | 4.33 | 48 |
| Budgie (Budgerigar) | 317 | 34% | 4.32 | 41 |
| Sparrow | 302 | 32% | 4.31 | 54 |
| Cockatoo | 271 | 29% | 4.76 | 35 |
| Hawk | 222 | 24% | 5.65 | 14 |
| Seagull | 196 | 21% | 5.82 | 12 |
| Emu | 194 | 21% | 5.63 | 28 |
| Dove | 193 | 21% | 4.62 | 26 |
| Kookaburra | 182 | 19% | 4.81 | 13 |
| Duck | 177 | 19% | 6.31 | 10 |
| Chicken | 175 | 19% | 6.37 | 14 |
| Owl | 173 | 19% | 4.90 | 31 |
| Finch | 161 | 17% | 5.11 | 18 |
| Swan | 151 | 16% | 6.21 | 7 |
| Canary | 139 | 15% | 3.93 | 40 |
| Galah | 131 | 14% | 5.54 | 10 |
| Robin | 116 | 12% | 3.57 | 42 |
| Pelican | 106 | 11% | 6.53 | 6 |
| Ostrich | 103 | 11% | 6.74 | 3 |
| Wren | 101 | 11% | 4.68 | 16 |
| Raven | 101 | 11% | 5.60 | 13 |
| Swallow | 98 | 10% | 4.73 | 15 |
| Penguin | 97 | 10% | 6.44 | 6 |
| Lorikeet | 84 | 9% | 5.58 | 8 |
| Ibis | 74 | 8% | 7.09 | 3 |
| Peacock | 68 | 7% | 5.87 | 6 |
| Goose | 67 | 7% | 7.52 | 1 |
| Hummingbird | 63 | 7% | 6.16 | 5 |
| Blackbird | 62 | 7% | 5.44 | 4 |
| Cockatiel | 61 | 7% | 5.72 | 5 |
| Falcon | 53 | 6% | 6.02 | 3 |
| Flamingo | 52 | 6% | 6.83 | 5 |
| Starling | 48 | 5% | 5.94 | 6 |
| Miner bird | 48 | 5% | 6.19 | 1 |
| Albatross | 45 | 5% | 7.44 | 1 |
| Rosella | 43 | 5% | 5.51 | 3 |

# Musical Instrument

Total participants: 978

Category size: 165

Mean number of responses: 8.34

Best category exemplars (italicised): Guitar, Piano

| **Instrument** | **Count** | **Production Frequency** | **Mean Rank** | **Number First** |
| --- | --- | --- | --- | --- |
| *Guitar* | *807* | *83%* | *2.76* | *254* |
| *Piano* | *789* | *81%* | *3.17* | *245* |
| Violin | 629 | 64% | 4.44 | 94 |
| Trumpet | 558 | 57% | 4.91 | 76 |
| Flute | 552 | 56% | 5.18 | 48 |
| Drums (Drum kit) | 496 | 51% | 4.49 | 59 |
| Saxophone | 341 | 35% | 5.37 | 16 |
| Drum | 304 | 31% | 3.59 | 62 |
| Cello | 294 | 30% | 6.16 | 10 |
| Trombone | 239 | 24% | 6.26 | 18 |
| Clarinet | 204 | 21% | 6.23 | 5 |
| Triangle | 198 | 20% | 7.58 | 1 |
| Harp | 194 | 20% | 6.58 | 13 |
| Bass | 190 | 19% | 6.33 | 1 |
| Oboe | 166 | 17% | 5.72 | 25 |
| Organ | 162 | 17% | 6.08 | 3 |
| Keyboard | 136 | 14% | 6.32 | 0 |
| Viola | 129 | 13% | 6.33 | 2 |
| Recorder | 123 | 13% | 7.09 | 2 |
| Cymbal | 119 | 12% | 7.78 | 0 |
| Tuba | 117 | 12% | 5.90 | 11 |
| Banjo | 104 | 11% | 5.75 | 7 |
| Xylophone | 87 | 9% | 8.34 | 2 |
| Double bass | 85 | 9% | 8.01 | 0 |
| Harmonica | 80 | 8% | 7.38 | 0 |
| Ukulele | 68 | 7% | 7.25 | 0 |
| Tambourine | 63 | 6% | 7.03 | 1 |
| Piccolo | 63 | 6% | 7.94 | 0 |
| Horn | 57 | 6% | 5.44 | 5 |
| Bass guitar | 52 | 5% | 6.31 | 3 |
| Bassoon | 47 | 5% | 7.47 | 0 |
